# Midlife Chronological and Endocrinological Transitions in Brain Metabolism: System Biology Basis for Increased Alzheimer’s Risk in Female Brain

**DOI:** 10.1038/s41598-020-65402-5

**Published:** 2020-05-22

**Authors:** Yiwei Wang, Yuan Shang, Aarti Mishra, Eliza Bacon, Fei Yin, Roberta Brinton

**Affiliations:** 1 Center for Innovation in Brain Science and Department of Pharmacology, University of Arizona, Tucson, AZ USA; 2 School of Pharmacy, University of Southern California, Los Angeles, CA USA

**Keywords:** Ageing, Metabolism, Mitochondria, Menopause

## Abstract

Decline in brain glucose metabolism is a hallmark of late-onset Alzheimer’s disease (LOAD). Comprehensive understanding of the dynamic metabolic aging process in brain can provide insights into windows of opportunities to promote healthy brain aging. Chronological and endocrinological aging are associated with brain glucose hypometabolism and mitochondrial adaptations in female brain. Using a rat model recapitulating fundamental features of the human menopausal transition, results of transcriptomic analysis revealed stage-specific shifts in bioenergetic systems of biology that were paralleled by bioenergetic dysregulation in midlife aging female brain. Transcriptomic profiles were predictive of outcomes from unbiased, discovery-based metabolomic and lipidomic analyses, which revealed a dynamic adaptation of the aging female brain from glucose centric to utilization of auxiliary fuel sources that included amino acids, fatty acids, lipids, and ketone bodies. Coupling between brain and peripheral metabolic systems was dynamic and shifted from uncoupled to coupled under metabolic stress. Collectively, these data provide a detailed profile across transcriptomic and metabolomic systems underlying bioenergetic function in brain and its relationship to peripheral metabolic responses. Mechanistically, these data provide insights into the complex dynamics of chronological and endocrinological bioenergetic aging in female brain. Translationally, these findings are predictive of initiation of the prodromal / preclinical phase of LOAD for women in midlife and highlight therapeutic windows of opportunity to reduce the risk of late-onset Alzheimer’s disease.

## Introduction

Late onset Alzheimer’s disease (LOAD) is a complex disease with approximately a 20-year prodromal period^1–3^. The prodromal/preclinical phase of AD is associated with brain glucose hypometabolism, which can be detected in at-risk-groups before diagnosis of the disease, and is predictive of disease progression^4–13^.

Brain glucose hypometabolism, mitochondrial dysfunction, and reduced oxygen flow in the brain are considered primary risk factors for LOAD^14–18^. On the cellular level, aging is associated with reduced glucose transporter expression, compromised hexokinase activity, phosphorylated (inactivated) PDH, and altered levels and activities of key enzymes involved in oxidative phosphorylation^19–36^. On the molecular level, aging is associated with significant down regulation of nuclear encoded OXPHOS genes^19,37^ and disrupted balance of NAD/NADH, AMP/ATP, purine and pyrimidine pool^38,39^.

During midlife, females experience both chronological and endocrinological aging. The perimenopausal to menopausal transition is unique to females, and is linked to deficits in brain glucose metabolism and mitochondrial dysfunction^3,19,40^, which could contribute to the two-fold greater lifetime risk of AD in females^41–43^.

Under normal conditions, brain utilizes glucose as its primary fuel source. Under stress conditions, the brain can adapt to utilize auxiliary fuel sources in response to an energy crisis. Under conditions of restricted nutrient access, auxiliary energy substrates such as lactate and ketone bodies are used to generate ATP^44–47^. Our previous research demonstrated that ketone bodies derived from brain lipids and white matter are utilized as an auxiliary fuel in the aging female brain in response to deficits in glucose metabolism^22,33^.

Herein, we describe the dynamic metabolic profile at each stage of midlife chronological and endocrinological aging (menopausal transition) in the female brain. Given the central role of mitochondria and electron transport chain (ETC) in brain bioenergetics, we investigated the transcriptome of both mitochondrial and nuclear encoded OXPHOS genes, followed by expression of key metabolic upstream regulators. We then detailed the dynamic metabolic profile in the female brain at each stage of aging, employing global metabolomics and lipidomic analysis, and supported these findings with transcriptomic analysis. Further, we characterized the relationship between brain and peripheral metabolic and lipid profiles.

## Materials and Methods

### Animals (Perimenopausal Animal Model of Human Menopausal Transition (PAM))

All animal studies were performed following National Institutes of Health guidelines on use of laboratory animals and all protocols were approved by the University of Southern California Institutional Animal Care and Use Committee.

To model human menopausal transition that included chronological and endocrinological aging, female Sprague Dawley rats were obtained from Harlan Laboratories (now part of Envigo) at either 5-month or 8-month of age. Their estrous cycle status was monitored and evaluated by vaginal cytology obtained through daily vaginal cytology between 9am to 11am over the course of 2 months. Details of the procedure and classification of estrous stages were previously described in detail by Yin *et al*.^19^. Briefly, the 4 stages of estrous cycle – estrus (E), metestrus (M), diestrus (D), and proestrus (P) – were morphologically characterized based on by the proportion of different cell types (epithelial cells, cornified cells, and leukocytes) presented in the vaginal secretions^19,48^. Female Sprague Dawley rats normally cycle through the four stages of estrous cycle in 4 to 5 days (regular). As they age, their reproductive system becomes incompetent, and the estrous cycle becomes unpredictable and prolonged, usually between 6 to 9 days (irregular), before they finally become reproductive senescent and stay constantly in the estrous stage (acyclic). This transition occurs in rats at around 9-month to 10-month of age^19,48^.

To capture this endocrinological transition, we included 9-10 months old regular cycling rats (Reg 9 mo), 9-10 months old irregular cycling rats (Irreg 9 mo), and 9-10 months old acyclic rats (Acyc 9 mo). And to test for age effect, we also included 6 months old regular cycling rats (Reg 6 mo) and 15 months old acyclic rats (Acyc 15 mo). To eliminate confounding effect of estrous cycle, all animals were euthanized on the estrous day of their estrous cycle. Rats that did not meet the endocrine status criteria were excluded from the study. For each assay, and an N of 5-7 per group was used.

Our initial study using the perimenopausal animal model (PAM) established validity of this model with regard to peripheral and brain female hormone levels^19^. Estrogen (E2) and progesterone (P4) levels were quantified by LC-MS/MS in serum and cerebral cortex collected at estrus across all groups^19^. We observed no correlation between serum and cortical E2 level at any given endocrine stage^19^. Serum E2 level was the highest in the Reg 9 mo and Irreg 9 mo group and declined in Acyc 9 mo and Acyc 15 mo^19^. In the cortex, E2 level dropped to negligible level from Acyc 9 mo to Acyc 16 mo^19^. In contrast to E2, serum P4 levels were correlated with cortical levels^19^. Serum P4 level was highest in the Irreg 9 mo group and significantly declined with the menopausal transition in the Acyc 9 mo group and remained low in the Acyc 16 mo group^19^. Similarly, cortical P4 level significantly decreased following transition from Irreg 9 mo to Acyc 9 mo^19^.

### Dissection of the brain

Rats were euthanized per animal protocol at University of Southern California, and brains were dissected quickly on ice to prevent degradation. Briefly, meninges were completely removed, followed by removal of hypothalamus, cerebellum, and brain stem. The two hemispheres were then separated, and hippocampus was peeled off from each hemisphere carefully. Brain tissues were snap frozen in liquid nitrogen before being stored in −80 °C for subsequent assays.

### RNA isolation

RNA was isolated following procedures previously described^49,50^. Briefly, frozen hippocampus tissue was directly homogenized in TRIzol Reagent (Invitrogen, 15596026) using the Bullet Blender and silicon beads. Chloroform was used to extract RNA from the homogenate at a volume ratio of 1:5 to that of the TRIzol Reagent. Ethanol was then used to precipitate nucleic acids from the aqueous phase. RNA was further purified using PureLink RNA Mini Kit (Invitrogen, 12183018 A) following manufacturer’s instructions. Purelink DNase (Invitrogen, 12185010) was used to eliminate DNA contamination. Purified RNA was eluded in RNase-free, diH_2_O. RNA concentration and quality were checked by NanoDrop One.

### RNA Sequencing (RNA-Seq)

RNA-Seq was conducted on hippocampal RNA at Vanderbilt Technologies for Advanced Genomics (VANTAGE). Only RNA samples with an acceptable RNA quality indicator score (RQI > 7) were used for sequencing. Enrichment of mRNA and library preparation of cDNA were done using a stranded mRNA (poly(A) - selected) sample preparation kit. Sequencing was performed at 100 bp paired-end on NovaSeq. 600, targeting 30 million reads per sample. Transcripts were mapped to rat genome (ensemble release 90) using Kallisto 0.4.3^51^. Tximport V1.6.0^52^ was used to generate a counts table from Kallisto output, and DESeq. 2 V1.18.1^53^ was used to calculate normalized read counts for each gene and/or transcript and to perform expression analysis. Heatmap visualization was based on average expression value of vst transformed normalized counts from DESeq. 2 and scaled per gene. N of 5-7 animals was included per group.

### Principle component analysis

The gene counts were transformed in the “DESeq. 2” package in R by variance stabilizing transformation. Then, PCAs were computed based on the top 500 variable genes. Projections over the first and second principal components were used to present the separations. To better visualize the phenotypic separation, the average PCs ± SEM for each group were calculated and presented in the PCA plot.

### Metabolome analysis

Changes in metabolomic profile during chronological and endocrinological aging processes was determined at each aging and endocrinological transition point. Both cortex (200μg) and plasma (100μL) samples were included to identify differences and correlations between the central nervous system and the peripheral system. Metabolomics analysis was performed by Metabolon utilizing their Global Metabolomics platform and the Complex Lipids Panel to identify changes in metabolic pathways. Briefly, the Global Metabolomics Platform used mass spectrometry to identify and quantify 438 compounds of known identity, covering classes of metabolites including amino acids, carbohydrates, lipids, nucleotides, microbiota metabolism, cofactors and vitamins, and xenobiotics. The Complex Lipids Panel focuses on the lipidomic, and determines absolute quantitation, molecular species concentration, and complete fatty acid composition of 990 named biochemicals from 14 lipid classes, including principle phospholipid, sphingolipid and neutral lipid classes. For heatmap visualization, normalized and log transformed data were scaled per metabolite. The distance maps of correlation between brain and plasma lipid profiles were generated using Orange 3.22 distance map feature without clustering. N of 5-7 animals was included per group.

### Ingenuity pathway analysis (IPA) of metabolomic data

Output of Metabolome data was processed using the metabolome analysis function of IPA. Because the nature of subtle changes during normal aging, metabolites with p value smaller than 0.25 were included. The disease and functions analysis predicted activation or inhibition of metabolic processes based on metabolites and networks compiled from literature and IPA’s Ingenuity knowledge base.

### Statistical analysis

For metabolomic analysis, mass spectrometry readings were normalized to mass and log transformed. Missing values were imputed with the minimum observed value of each compound. Welch’s two-sample *t*-test was used to identify biochemicals that differed significantly between experimental groups. For the lipidomic correlational analysis between brain and plasma, Pearson correlation was used to calculate distance matrix. A p value of <0.05 is considered statistically significant for all statistical analysis in this study.

## Results

### Hippocampal transcriptome

**Figure 1:**
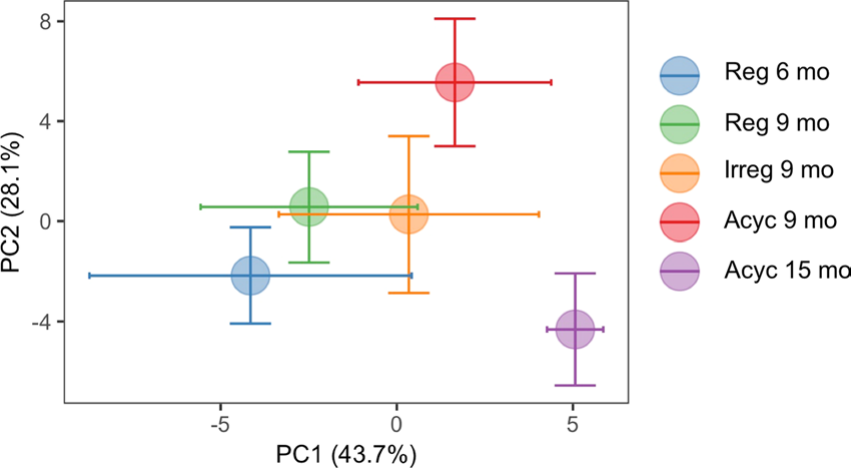
Principle component analysis of hippocampal gene expression. PC1 can be explained by both chronological aging and endocrinological aging.  Chronological aging has a stronger impact on the trancriptomic profile, especially post-menopausal,  which led to a unique aging brain state.

RNA-Seq analysis of bulk hippocampal gene expression and principle component analysis revealed distinct transcriptional profiles as females transitioned through chronological and endocrinological aging (Fig. 1). Principle component analysis indicated that up to 43.7% of variance in the transcriptome was explained by chronological aging and endocrinological aging combined (PC1), and chronological aging had a stronger impact on hippocampal transcriptome than endocrinological aging (Fig. 1 and Table 1). Further, chronological aging had more impact during post-menopausal aging, between 9-month-old animals and 15-month-old animals, than during pre-menopausal aging, between 6-month-old animals and 9-month old animals (Fig. 1 and Table 1). PCA also suggested that during endocrinological aging, Acyc 9 mo animals that completed the menopausal transition had distinct transcriptomic profile relative to reproductively competent Reg 9 mo animals and reproductively impaired Irreg 9 mo animals (Fig. 1). These observations confirmed our previous findings in hypothalamus, where changes in gene expression and DNA methylation profiles precedes reproductive senescence and changes in the hippocampus^54^.

**Table 1:** Number of differentially expressed genes (DEGs, p < 0.05) between each chronological or endocrinological aging transition stage. Post-menopausal aging is associated with the greatest number of DEGs.

	Reg 9 mo vs Reg 6 mo	Irreg 9 mo vs Reg 9 mo	Acyc 9 mo vs Irreg 9 mo	Acyc 15 mo vs Acyc 9 mo
Upregulated	207	144	269	2285
Down-regulated	159	219	477	1888

### Brain bioenergetics gene expression

**Figure 2:**
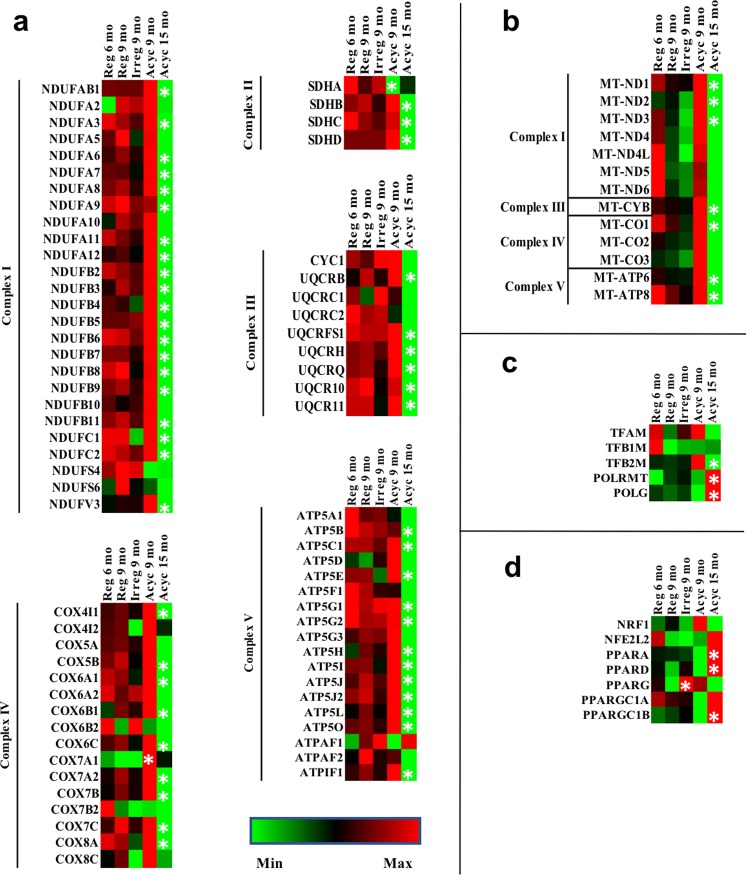
Gene expression of hippocampal electron transport chain subunits and bioenergetics regulators during endocrinological and chronological aging. Panel (a), nuclear encoded OXPHOS genes; (**b**), mitochondrial encoded OXPHOS genes; (**c**), expression of mitochondrial transcriptional factors, RNA polymerase POLRMT and DNA polymerase POLG; (**d**), gene expression of key bioenergetics regulators. *p < 0.05 in comparison to the previous column.

Consistent with the overall trend in the hippocampal transcriptome, RNA-Seq analysis revealed that chronological aging had greater impact on oxidative phosphorylation (OXPHOS) gene expression, especially between Acyc 9 mo and Acyc 15 mo (Fig. 2). Multiple subunits encoded by both nuclear (Fig. 2a) and mitochondrial genome (Fig. 2b), across all five electron transport chain complexes, were significantly down-regulated. During endocrinological aging, animals displayed an initial trend of decline in OXPHOS gene expression at Irreg 9 mo and a non-significant rebound as the transition completed at Acyc 9 mo, for both nuclear and mitochondrial encoded subunits (Fig. 2a and Fig. 2b).

The pattern of expression of mitochondrial transcriptional factor TFB2M was significantly down-regulated during post-menopausal chronological aging (p = 0.015), and TFAM displayed a trend towards down-regulation, supporting the observed change in mitochondrial gene expression (Fig. 2c). In contrast, mitochondrial RNA polymerase (POLRMT) and DNA polymerase (POLG) were significantly upregulated during post-menopausal aging (p = 0.014 and p = 0.007 respectively), which could be interpreted as a compensatory response to decline in TFAM (p = 0.056), TFB2M, and mitochondrial gene expression (Fig. 2c).

Given the changes in gene expression of electron transport chain subunits, we investigated gene expression of key energy metabolism and bioenergetic regulators (NRF1, NFE2L2, PPARA, PPARD, PPARG, PPARGC1A, PPARGC1B). RNA-Seq revealed that chronological aging from Reg 6 mo to Reg 9 mo was associated with a trend towards down-regulation of these genes, whereas chronological aging from Acyc 9 mo to Acyc 15 mo was associated with significant upregulation of multiple bioenergetic genes, including PPARA (p = 0.023), PPARD (p = 0.004), and PPARGC1B (0.008) (Fig. 2d). The endocrinological aging process was associated with a temporary surge of expression of energy metabolism regulators, especially PPARG, at Irreg 9 mo (Fig. 2d). Given the role of these regulatory genes in glucose and lipid metabolism, fluctuating gene expression patterns suggested potential dynamic fuel changes in the aging female brain, which we then investigated.

### Brain metabolome

#### Glycolysis and TCA cycle

**Figure 3:**
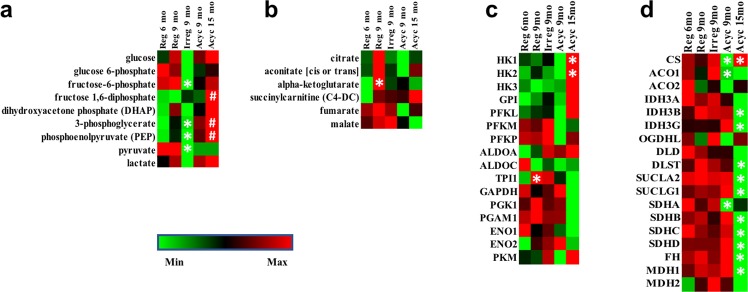
Glycolysis and TCA cycle intermediates and key enzyme gene expression. Panel (a), glycolysis intermediates and lactate level; (**b**), TCA cycle intermediates and derivatives level; (**c**), gene expression of key enzymes involved in glycolysis; (**d**), gene expression of key enzymes involved in TCA cycle. *p < 0.05 in comparison to the previous column. ^#^p < 0.05 in comparison to Reg 6 mo.

Metabolomic analysis in brain indicated that compared to the Reg 6 mo group, Reg 9 mo group had significantly higher level of α-ketoglutarate (p = 0.033), along with multiple other TCA cycle intermediates and glycolysis intermediates (citrate, aconitate, dihydroxyacetone phosphate, 3-phosphoglycerate, and phosphoenolpyruvate). Compared to the Reg 9 mo group, Irreg 9 mo group had a significant reduction in multiple glycolysis intermediates (frugcose-6-phosphate (p = 0.035), 3-phosphoglycerate (p = 0.046), phosphoenolpyruvate (p = 0.029), and pyruvate (p = 0.021), Fig. 3a), with a trend towards reduction in TCA cycle intermediates (citrate, aconitate, and α-ketoglutarate, Fig. 3b). This observation was consistent with our previous findings of significantly reduced brain glucose uptake and electron transport chain complexes I and IV activities during the same stage of endocrine aging^19^. Further, compared to the Reg 6 mo group, Acyc 15 mo had significantly higher glycolysis intermediates (fructose 1,6-diphosphate (p = 0.006), 3-phosphoglycerate (p = 0.008), and phosphoenolpyruvate (p = 0.005), Fig. 3a). However, Acyc 15 mo group had reduced pyruvate (Fig. 3a) and TCA cycle intermediates (Fig. 3b). This can be explained by the significant down-regulation of genes encoding TCA cycle enzymes (IDH3B, IDH3G, DLST, SUCLA2, SUCLG1, SDHB, SDHC, SDHD, FH, and MDH1), oxidative phosphorylation genes (Fig. 3), and a trend towards decreased expression of glycolysis genes (Fig. 3d). Hexokinase 1 and 2 were significantly upregulated, suggesting a futile compensatory response (Fig. 3c).

#### Amino acid and peptide metabolism

**Figure 4:**
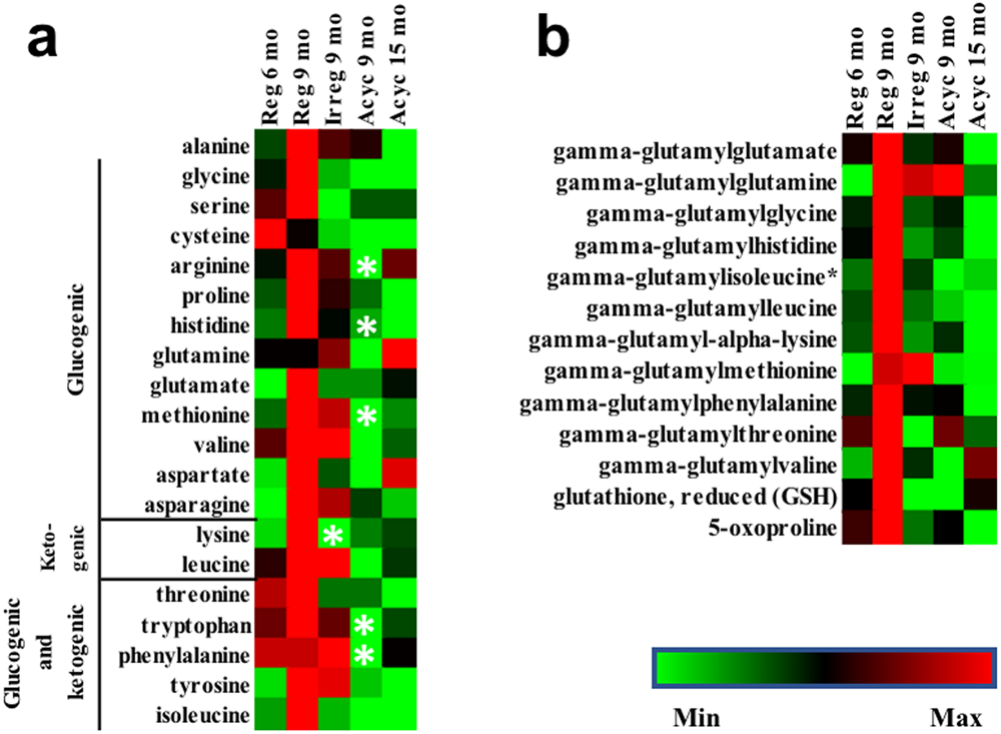
Brain amino acids levels (**a**) and gamma-glutamyl amino acids levels (**b**). *p < 0.05 compared to Reg 9 mo group.

While brain relies on glucose as its primary fuel source, auxiliary fuels such as amino acids, fatty acids, and ketone bodies can be used in response to changing fuel source availability. For this reason, we investigated whether fluctuating glycolysis and TCA cycle activity affected amino acid metabolism. Brain amino acid levels peaked in Reg 9 mo (Fig. 4a). Coupled with the highest levels of gamma-glutamyl amino acids, reduced glutathione (GSH), and 5-oxoproline at this stage (Fig. 4b), which are involved in transporting amino acids across cellular membrane, these data suggested increased amino acid metabolism in reg 9 mo group. IPA metabolomic pathway analysis predicted significant activation of amino acid release during this stage (z = 2.327, p = 2.06e-8). Because glucogenic amino acids (Ala, Arg, Asn, Asp, Cys, Glu, Gln, Gly, His, Met, Pro, Ser, Val, Phe, Ile, Thr, Trp, Tyr) can feed into the TCA cycle as intermediates, the elevated amino acid level provided a plausible explanation for the trend toward increase of multiple TCA cycle intermediates, especially α-ketoglutarate (p = 0.03) (Fig. 3b). Oxaloacetate from the TCA cycle can be further converted to phosphoenolpyruvate for gluconeogenesis. However, due to the extremely low gene expression level of fructose-bisphosphatase in the brain, the enzyme catalyzing the rate limiting step that converts fructose-1,6-diphosphate to fructose 6-phosphate, carbon backbone of amino acids is unlikely to be converted to fructose-1,6-biphosphate for glucose production. This fits well with the non-significant increase of phosphoenolpyruvate, 3-phosphoglycerate and DHAP and no increase in fructose-1,6-diphosphate, fructose-6-phosphate, or glucose-6-phosphate in the Reg 9 mo group (Fig. 3a).

#### Ketone body metabolism

**Figure 5:**
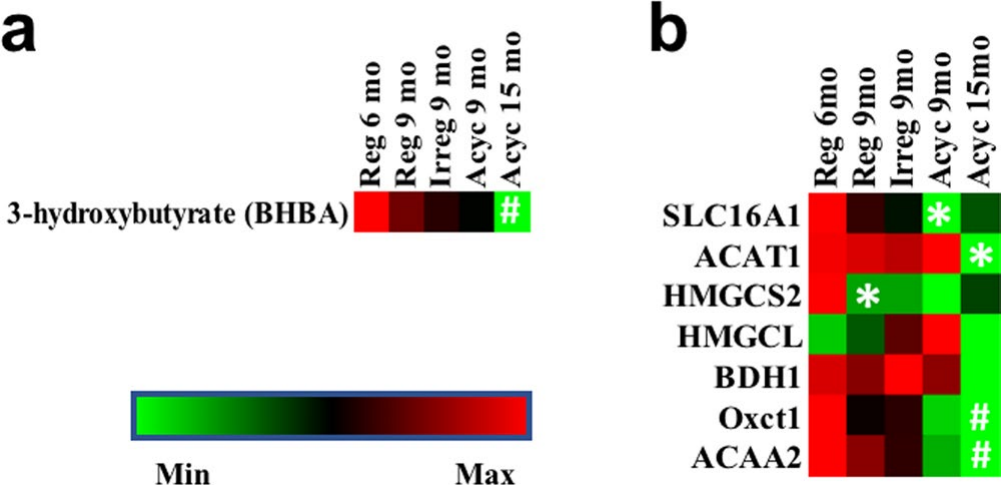
Brain ketone body level (**a**) and expression of genes involved in ketone body metabolism (**b**). *p < 0.05 in comparison to the previous column. ^#^p < 0.05 in comparison to Reg 6 mo.

Because ketogenic amino acids (Lys, Leu, Thr, Tyr, Phe, Trp, Ile) can be used to generate ketone bodies, we investigated the metabolic profile of ketone body generation. Metabolomic analysis revealed a gradual decline of 3-hydroxybutyrate during chronological and endocrinological aging (p = 0.021, Fig. 5a). Transcriptome analysis confirmed that genes for ketone body transport (SLC16A1), ketogenesis (ACAT1, HJGCS2, HNGCL, BDH1), and ketolysis (BDH1, OXCT1, ACAA2) all declined with chronological and endocrinological aging (Fig. 5b). These data suggest that the surge of amino acid metabolism in the Reg 9 mo group was not associated with ketone body metabolism.

#### Glycolipids metabolism

**Figure 6:**
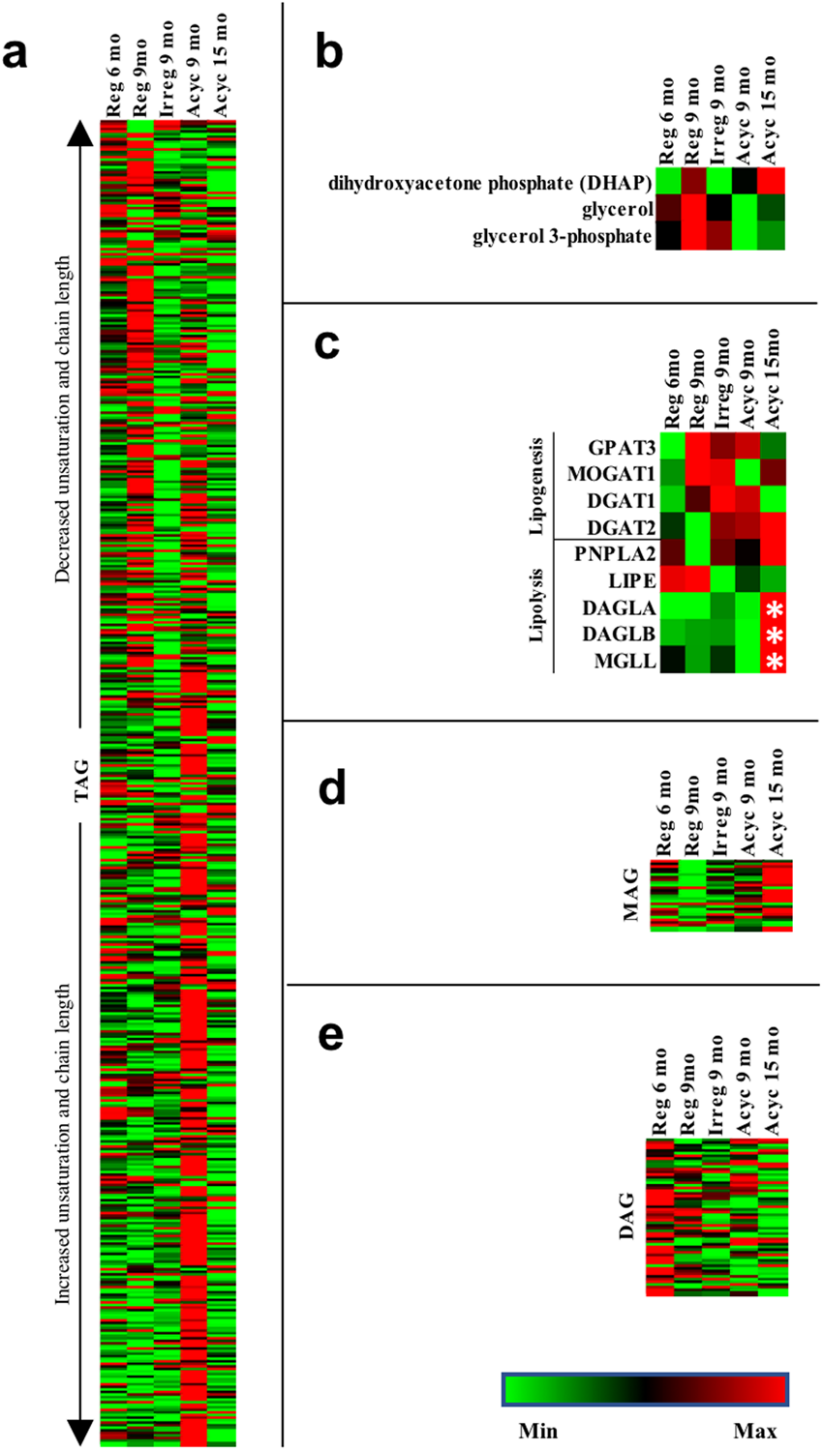
Glycolipids metabolism and expression of relevant genes. Panel (a), brain triglyceride levels; (**b**), DHAP, glycerol, and glycerol 3-phosphate levels; (**c**), gene expression of lipogenesis and lipolysis enzymes; (**d**), brain monoacylglycerol levels; (**e**), brain diacylglycerol levels. Lipids arranged by increased unsaturation and total carbon number from top to bottom for panels (a,d,e). *p < 0.05 compared to the previous column.

Because the systematic shift in amino acid metabolism and TCA cycle intermediates did not lead to gluconeogenesis or ketogenesis, we hypothesized that these changes may instead lead to lipogenesis. Indeed, IPA metabolomic analysis predicted activation of re-esterification of lipids (z = 2.00, p = 9.58e-7) from Reg 6 mo to Reg 9 mo. Further, change in triglyceride (TAG) level was fatty acid side chain saturation-dependent. In general, levels of TAGs with saturated or monounsaturated fatty acid side chains were the highest in the Reg 9 mo group, in contrast to those with polyunsaturated side chains (Fig. 6a). Transcriptomic analysis suggested two potential mechanisms for lipogenesis at Reg 9 mo. The first route was *de novo* synthesis from DHAP. We observed significant upregulation of the TPI1 gene, which encodes the enzyme that catalyzes the interconversion of glycolysis intermediate Glyceraldehyde-3-phosphate to DHAP, the precursor to triglycerides (Fig. 3c). This observation is further supported by increased levels of DHAP, glycerol, and glycerol 3-phosphate (Fig. 6b), and non-significant upregulation of GPAT3 (Fig. 6c), which catalyzes rate limiting step of glycerol-3-phosphate to lysophosphatidic acid conversion. The second route is conversion from monoacylglycerol (MAG) (Fig. 6d), and was supported by increased expression of MOGAT1 and DAGT1 (Fig. 6c), which are responsible for converting MAG to diacylglycerol (DAG) (Fig. 6e) and DAG to TAG respectively.

In contrast, post-menopausal aging from Acyc 9 mo to Acyc 15 mo was associated with significant activation of lipid catabolism (z = 2.376, p = 3.18e-6), especially polyunsaturated TAGs (Fig. 6a). This was evident by a significant upregulation of diacylglycerol lipase (DAGLA and DAGLB) and monoacylglycerol lipase (MGLL), and non-significant upregulation of triglyceride lipase (PNPLA2) (Fig. 6c). Increased lipolysis and potentially stalled β-oxidation in the Acyc 15 mo group could explain the accumulation of monoacylglycerol in this group.

#### Fatty acid metabolism

**Figure 7:**
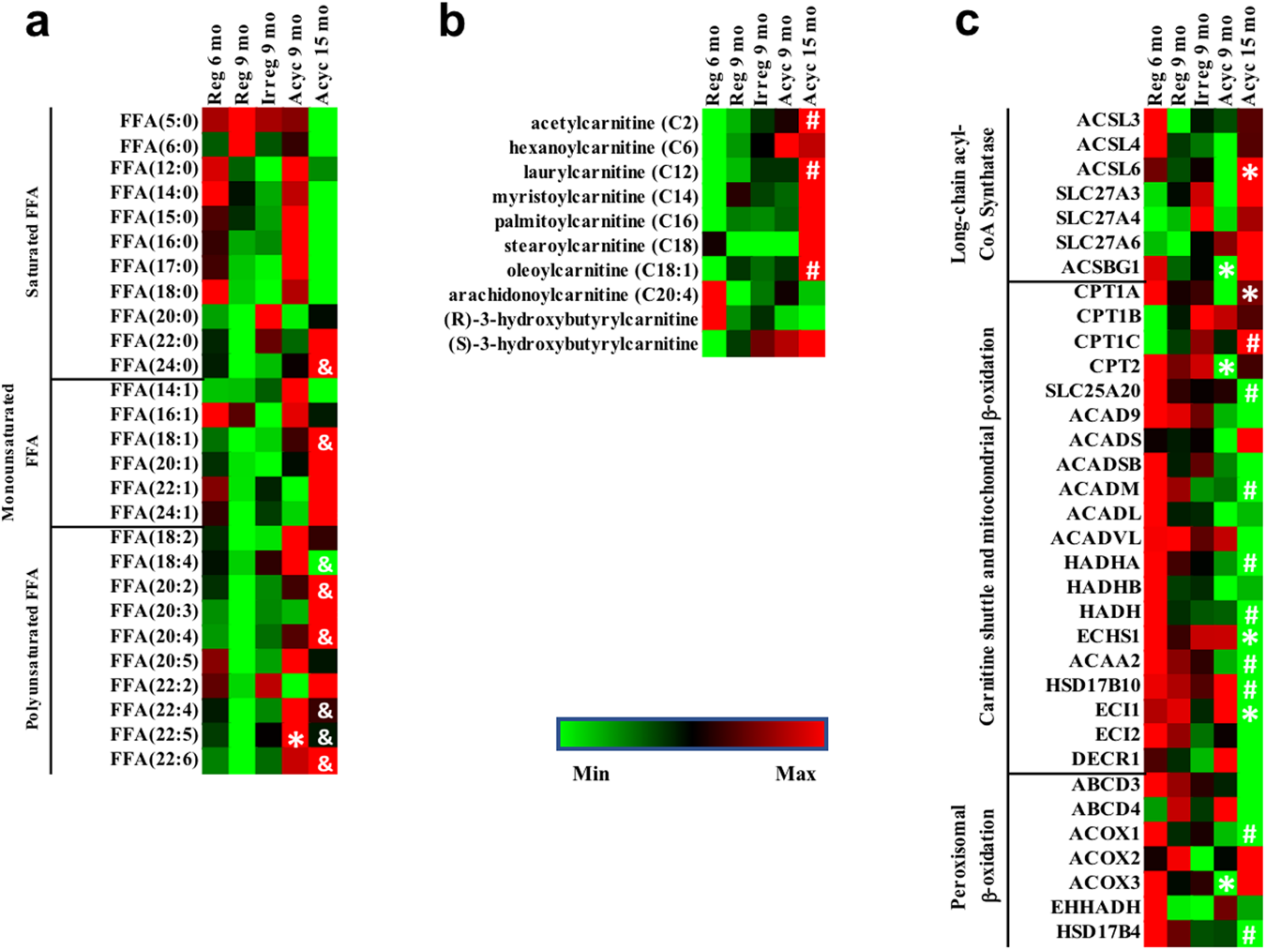
Fatty acid metabolism. Panel (a), level of free fatty acid (FFA) levels in the brain; (**b**), level of acyl-carnitine levels in the brain; (**c**), gene expression of genes involved in fatty acid metabolism and β-oxidation. *p < 0.05 compared to the previous column. ^#^p < 0.05 to Reg 6 mo group. ^&^p < 0.05 to Reg 9 mo group.

Fatty acids are major components of glycolipids. Systematic shifts in lipogenesis and lipolysis should be paralleled by shifts in fatty acid metabolism. Consistent with the pattern of TAG metabolism, fatty acid metabolism in the midlife aging female brain is also chain length- and saturation-dependent (Fig. 7). Saturated short-chain and long-chain fatty acid levels were the lowest in the Acyc 15 mo group. On the other hand, very long-chain fatty acids and unsaturated fatty acids were the lowest in the Reg 9 mo group and accumulated in the Acyc 15 mo group (Fig. 7a). Fatty acids can be catabolized through β-oxidation in either mitochondria or peroxisome. Post-menopausal aging from Acyc 9 mo to Acyc 15 mo was associated with upregulation of long-chain acyl-CoA synthetase (Fig. 7c), which enables long-chain fatty acids to be transported into the mitochondria or peroxisome, as indicated by a significant increase of lipid transportation (z = 2.635, p = 2.34e-15), and fatty acid activation (z = 2.333, p = 1.85e-11). However, while carnitine palmitoyl transferases (CPT1 and CPT2) were upregulated in the Acyc 15 mo group, the carnitine-acylcarnitine translocase (SLC25A20) was significantly down-regulated (Fig. 7c), indicating potential impairment of the carnitine shuttle, which is responsible for transferring acylcarnitine into mitochondria. Similarly, transporters responsible for importing long-chain fatty acids into the peroxisome (ABCD3, ABCD4) were also down-regulated, although changes were not statistically significant (Fig. 7c).

Coincident with observed changes in fatty acid transporters, multiple genes encoding enzymes for β-oxidation in both mitochondrial and peroxisome were down-regulated (Fig. 7c). These alternations of genomic responses could explain the downregulation of fatty acid beta oxidation (z = -0.853, p = 1.27e-26) and the accumulation of acylcarnitine in the Acyc 15 mo group (Fig. 7b). Coupled with the inhibition of TCA cycle (z = 2.828, p = 2.59e-29), this observation further explained the down-regulation of mitochondrial OXPHOS genes (Fig. 2). However, since short-chain fatty acids can enter mitochondria without the carnitine shuttle, they still can be readily metabolized, which is consistent with the non-significant upregulation of ACADB, the acyl-CoA dehydrogenase that has specific activity for short-chain fatty acids (Fig. 7c). Because branched chain fatty acids are mostly saturated fatty acids, the non-significant upregulation of ACOX2 and ACOX3, two acyl-CoA oxidase isoforms involved in branched chain fatty acid metabolism in peroxisomes, is also consistent with the reduced saturated fatty acids level in the Acyc 15 mo group (Fig. 7).

#### Plasma lipidome

**Figure 8:**
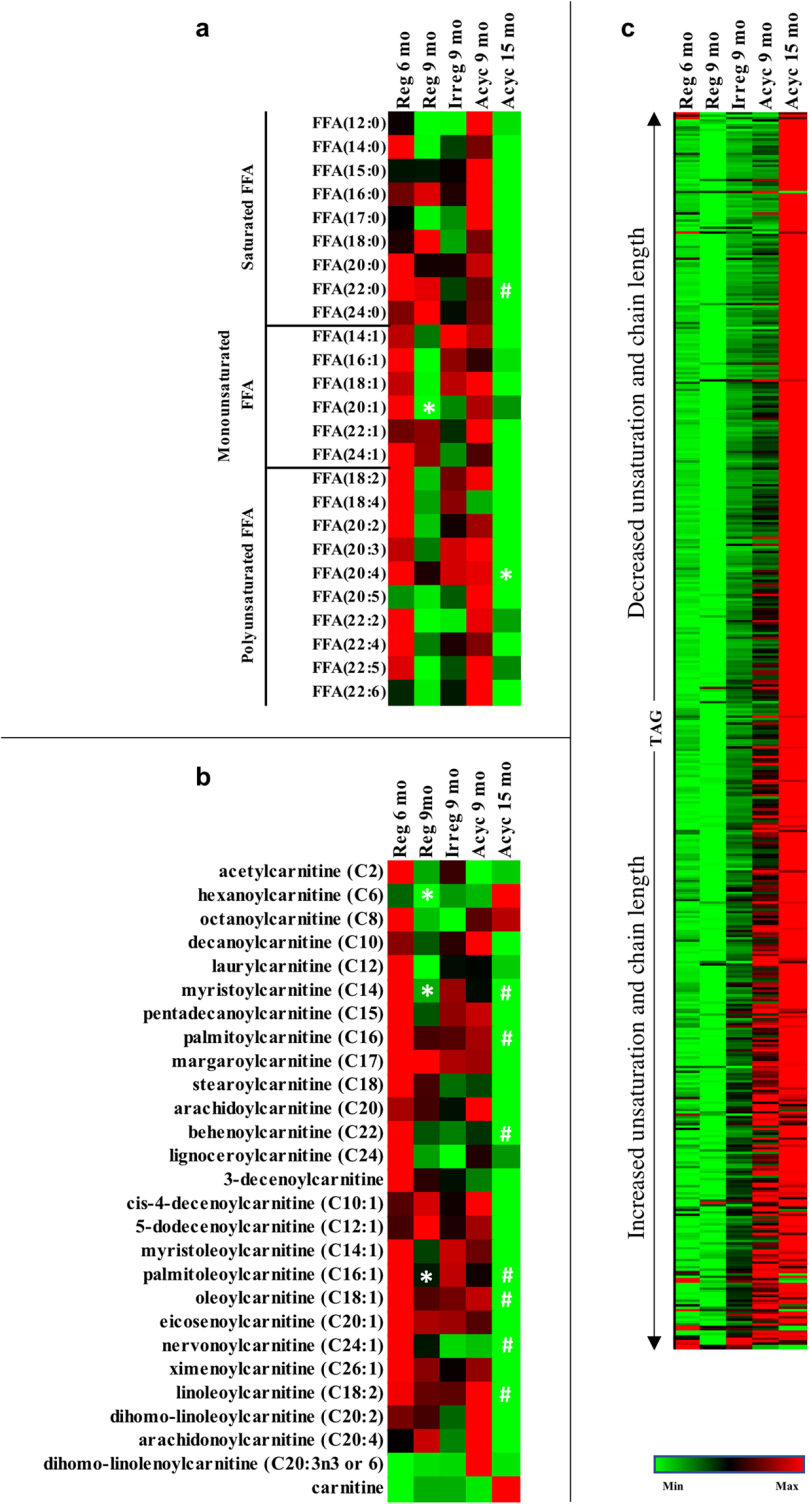
Plasma lipidomic analysis. Panel (**a**), free fatty acid levels; (**b**), acylcarnitine and free carnitine levels; (**c**), triglyceride (TAG) levels. *p < 0.05 to the previous column. ^#^p < 0.05 to Reg 6 mo.

The lipid metabolic profile in plasma was distinct from that of brain. In plasma, metabolomic analysis revealed a decline in free fatty acid levels in Reg 9 mo group (Fig. 8a). Unlike the brain, which released fatty acids from lipids, plasma fatty acids were likely converted to triglyceride for storage in Acyc 15 mo group, as evident by a decrease in free fatty acid levels (Fig. 8a) and increased triglyceride levels (p < 0.05 for 406 out of 510 TAG species compared to Reg 9 mo) (Fig. 8c). Significantly lower levels of multiple acyl carnitine and relatively higher free carnitine levels supported the hypothesis of reduced fatty acid utilization in the peripheral in Acyc 15 mo animals (Fig. 8b). In parallel with the brain, ketone body levels gradually decreased in plasma. The dynamic metabolic and lipidomic profiles highlighted changes in systems of biology indicative of transitioning from midlife aging to late life aging.

#### Brain-plasma lipid profile correlation

**Figure 9:**
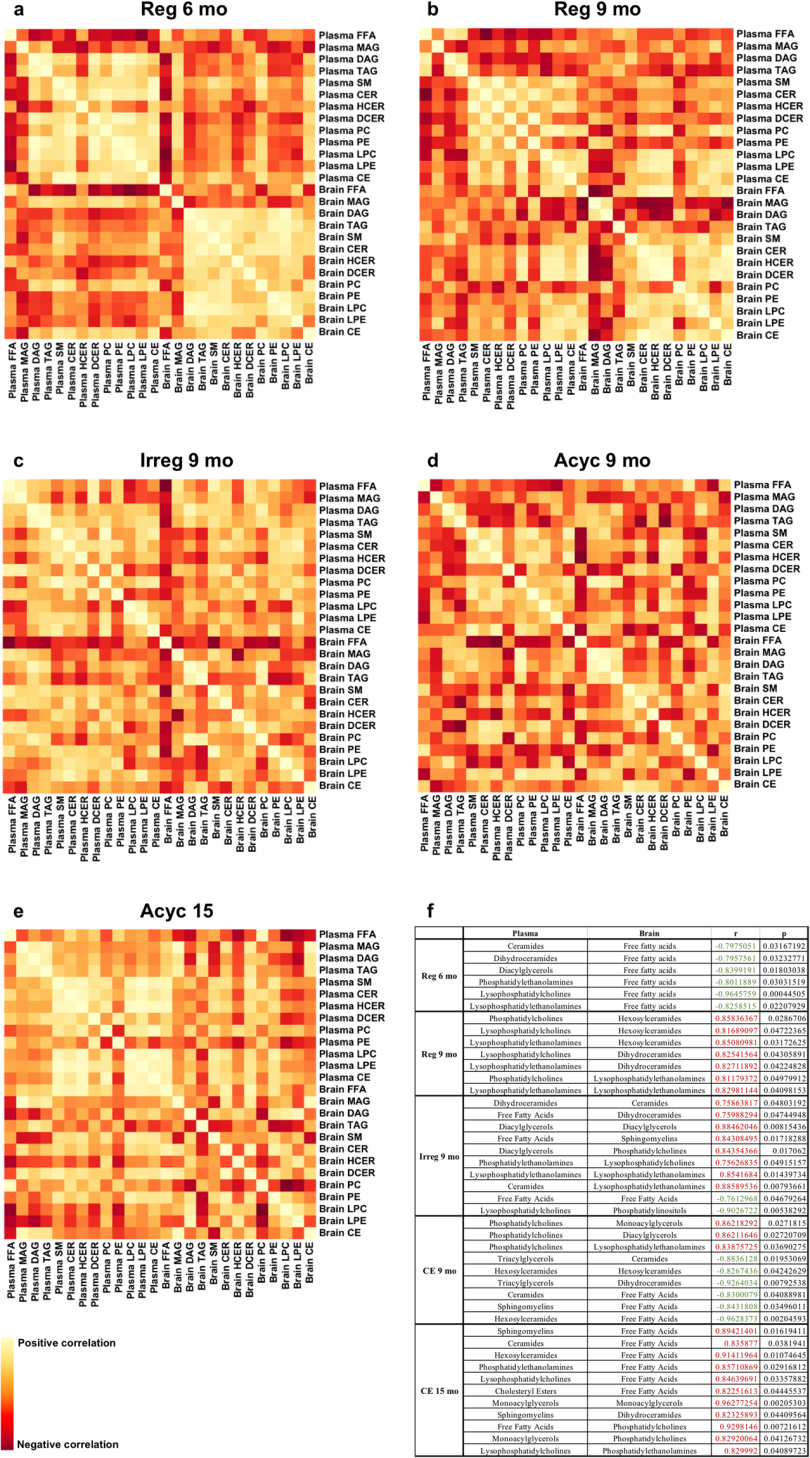
Correlation of brain and plasma lipid profiles at each chronological and endocrinological aging stage. Panel (**a**), Reg 6 mo; (**b**), Reg 9 mo; (**c**), Irreg 9 mo; (**d**), Acyc 9 mo; (**e**), Acyc 15 mo; (**f**), summary of significant correlations between brain and plasma lipid species. Dark red indicates negative correlation, and bright yellow indicates positive correlation. FFA, free fatty acid; MAG, monoacylglycerol; DAG, diacylglycerol; TAG, triacylglycerol; SM, sphingomyelin; CER, ceramide; HCER, hexosylceramid; DCER, dihydroceramide; PC, phosphatidylcholine; PE, phosphatidylethanolamine; LPC, lysophosphatidylcholine; LPE, lysophosphatidylethanolamine; CE, cholesterol ester.

Correlation analysis of brain and plasma lipidome revealed that the lipid metabolic profiles were dynamic within each compartment and independent of each other across chronological and endocrinological aging stages (Fig. 9). Significantly positive correlations among ceramides (CER), phosphatidylcholines (PC), phosphatidylethanolamines (PE), sphingomyelins (SM), diacylglycerols (DAG), and triacyclglycerols (TAG) were observed at 6 months within either brain or plasma (Fig. 9a). In contrast, an overall negative correlation was observed between the two systems, especially between brain total free fatty acid (FFA) and plasma ceramides, phospholipids, lysophospholipids, and diacylglycerols (Fig. 9a). These data suggested dynamic and independent lipid interconversion and homeostasis within each system in young and reproductive competent females.

As animals transitioned from Reg 6 mo to Reg 9 mo, correlation analysis revealed a pattern of declining positive correlation among lipid species within both brain and plasma, and more positive correlations between the two compartments, where the significant negative correlation between brain total free fatty acid and plasma lipids was lost (Fig. 9b). These observations suggested a potential transition from independent metabolic compartments to interdependence between the two systems. At this stage, brain free fatty acid (FFA) level was significantly negatively correlated with brain monoacylglycerols (MAG) and diacylglycerols (DAG) (Fig. 9b), consistent with our metabolomic and transcriptomic analysis suggesting sequestering of brain free fatty acids for lipogenesis. A similar pattern of free fatty acid sequestration was also observed in plasma, although fatty acids were negatively correlated with ceramides instead of glycolipids (Fig. 9b).

The shift in lipid metabolism continued into Irreg 9 mo, especially in the brain, where significant correlations among lipid species were limited to phosphatidylcholine, triacylglycerol, and diacylglycerols, and between sphingomyelin and phosphatidylethanolamine (Fig. 9c). In contrast, positive correlations remained among triacylglycerol, diacylglycerol, monoacylglycerol, and free fatty acids, as well as among phosphatidylcholine, ceramides, sphingomyelin, and cholesterol esters (CE) were present in plasma, (Fig. 9c). Further, brain free fatty acid was negatively correlated with most lipid species in both brain and plasma, including plasma free fatty acids (Fig. 9c).

At Acyc 9 mo, disruption in lipid metabolism was evident in both brain and the periphery. Very few significant correlations were observed within either system or between the two (Fig. 9d). In brain, the sole significant correlation was a positive correlation between monoacylglycerol and diacylglycerol (Fig. 9d). And in plasma, the sole significant correlation was a positive association among hexosylceramide (HCER), sphingomyelin, and cholesterol ester (Fig. 9d). Brain and plasma lipid species appeared to be negatively correlated again, particularly between brain free fatty acids and plasma ceramides and sphingomyelin, and between brain ceramides and plasma triglycerides (Fig. 9d).

At Acyc 15 mo, lipid homeostasis appeared to be restored among most plasma lipid species, but to a lesser degree in brain (Fig. 9e). In plasma, positive correlations were observed between triacylglycerol and diacylglycerol, triacylglycerol and monoacylglycerol, as well as among sphingomyelin, ceramide, cholesterol ester, and lysophospholipids (Fig. 9e). In the brain, ceramides were positively correlated with free fatty acids, as well as phosphatidylethanolamine level (Fig. 9e), suggesting potential *de novo* synthesis of ceramide in the brain, as observed in our previous studies^33^. In contrast to the negative correlation observed at Reg 6 mo, brain free fatty acid was significantly positively correlated with plasma cholesterol ester, ceramide, lysophospholipids, and sphingomyelin, but not plasma fatty acids (Fig. 9e).

## Discussion and Conclusion

Aging is the greatest risk factor for late onset AD^55^. While the average age of diagnosis for AD is in the mid 60 s to 70 s, a prodromal phase of 10 to 20 years precedes diagnosis^55–58^. The average age of menopause is ~51 years of age^59^, which is approximately 20 years prior to the average age of AD diagnosis. The coincidence of completion of the menopausal transition and the prodromal phase of AD in females may explain the higher risk for developing mild cognitive impairment between age 55 to 70 years^60^, higher risk for developing AD between age 65 to 75^60^, and a two-fold life-time risk of LOAD in comparison to males^43,61–63^.

To address change in bioenergetic function, we first investigated gene expression of both nuclear and mitochondrial encoded OXPHOS genes, and observed down-regulation of OXPHOS genes in Acyc 16 mo animals. Down-regulation of OXPHOS genes was paralleled by decline in mitochondrial transcriptional factors (TFAM, TFB1M, TFB2M). When OXPHOS gene transcription was down-regulated, PPARA and PPARD were upregulated, suggesting a compensatory response to promote mitochondrial biogenesis. Increased expression of PPARA and PPARD was paralleled by increases in mitochondrial DNA polymerase POLG and RNA polymerase POLRMT. Collectively, the data are consistent with a shift in patterns of both nuclear and mitochondrial gene expression that is paralleled by mitochondria phenotypes consistent with the transcriptional profile.

**Figure 10:**
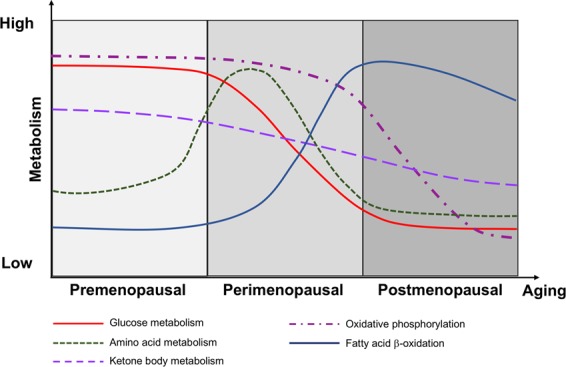
Dynamic metabolic aging in female brain during chronological and endocrinological aging. Decline in glucose metabolism results in utilization of amino acids as an interim metabolic fuel alternative, followed by activation of fatty acid metablism, as oxidative phosphorylation continues to decline.

The change in mitochondrial transcriptional profile was paralleled by adaptations in fuel supply (Fig. 10). We observed that different fuel sources were differentially preferred at each stage of aging, and that the perimenopausal transition was a turning point in brain bioenergetics and metabolic profile (Fig. 10). Prior to the onset of perimenopausal transition, the female brain primarily utilized glucose for fuel. Decline in glucose metabolism across the menopausal transition observed herein replicates our earlier findings in animal models^19,20,23,26^, which translated to human female brain^40,64^. Chronological aging from 6-months reproductively competent animals to 9-month reproductively competent animals was associated with increased utilization of amino acids as fuel sources. Given the lack of gluconeogenesis or ketogenesis observed in Reg 9 mo animals, the carbon backbone of amino acids was likely shuttled to DHAP for triglyceride production. At the same time, monoacylglycerols also tended to be converted to triglycerides, explaining the nonsignificant but systematic decrease of monoacylglycerol and free fatty acids which was paralleled by increased triglycerides, particularly saturated or monounsaturated triglycerides.

Onset of endocrinological transition to the perimenopause was characterized by a shift from amino acid metabolism to lipid metabolism, as amino acids no longer sustained the energetic demand of the brain. In parallel, glycolysis was impaired, as evident by significant reduction in multiple glycolysis intermediates. This is consistent with a trend towards down-regulation of OXPHOS genes observed in this study, as well as reduced glucose uptake, electron transport chain complex I and complex IV activities, and mitochondrial respiratory capacity as in our previous studies^19,20,23^.

During post-menopause chronological aging, mitochondria became progressively inefficient in oxidative phosphorylation and fatty acid beta oxidation. While short chain fatty acids can still enter mitochondria and be metabolized for energy production, long chain fatty acids could not be effectively transported into mitochondria, which led to accumulation of long-chain fatty acids (Fig. 7a). In a potential feedback mechanism, triglycerides can be continuously catabolized to generate free fatty acids. This observation is in drastic contrast to plasma, where triglycerides were significantly elevated during post-menopausal aging. The outcome of brain lipid catabolism could be two-fold. First, free fatty acids can stimulate acylcarnitine production, satisfying a key step in transporting long-chain acyl-CoA into mitochondria for β-oxidation while also serving as a marker of incomplete β-oxidation^65,66^. Second, as a feedback mechanism it could stimulate glucose metabolism as a compensatory mechanism, which would be consistent with upregulation of genes involved in glycolysis in aged reproductive senescent rats compared to newly menopausal rats. However, deficiency in TCA cycle and electron transport chain activity would restrict energy production through oxidative phosphorylation, thereby promoting anaerobic glycolysis in the brain (production of lactate rather than pyruvate). Throughout the whole transition, the level of ketone bodies gradually diminished, due to reduced ketogenesis, which is consistent with literature showing reduced ketone body metabolism with aging in human studies^67^.

In addition to their role as an auxiliary fuel source, lipids are also key components of cellular membranes and are involved in lipid storage and transportation. The observed alterations in lipid metabolism during chronological and endocrinological aging are consistent with our previous analysis demonstrating fluctuation in lipid droplet accumulation and increase in myelin degradation as an adaptive approach of the aging female brain to generate an alternative energy source^22,33^. The alteration in myelin composition resembles the human aging and AD metabolic phenotype of increased lipid metabolism and myelin turnover, with decreased energy metabolism and mitochondrial function^68,69^.

The lipidomic profile in plasma is consistent with a dynamic response to challenges in providing fuel for brain ATP generation. The dynamic response was particularly evident in free fatty acids early during both chronological (Reg 6 mo to Reg 9 mo) and endocrinological aging (Reg 9 mo to Irreg 9 mo). Completion of reproductive senescence was characterized by a shift in plasma metabolome from a glucose metabolomic phenotype to a fatty acid and ultimately a triglyceride lipid phenotype.

The correlational analysis of brain and plasma lipid profiles further revealed that while both systems shift from glucose metabolism to lipid metabolism, the dynamic changes were not completely paralleled, highlighting the complexity and multi-directionality of lipid interconversion within each system. During the transition from premenopausal chronological aging to perimenopausal endocrinological aging, lipid metabolic homeostasis within brain and plasma were gradually disrupted. Our analysis suggested that while the peripheral system could restore the balance to a younger phenotype during postmenopausal aging, the brain lipid metabolism remained disrupted. This is consistent with our brain metabolic and transcriptomic analysis suggesting that post-menopausal brains had limited capacity for both oxidative phosphorylation and β-oxidation.

While this study focused on bioenergetic and metabolic aging in female brain, the underlying mechanisms reported herein may be relevant to aging in the males. Compared to menopause, andropause is a similar transition with a wider age window, longer duration, and more gradual loss of sex hormones. The aging male brain also displays glucose hypometabolism^70,71^, which is correlated with waning level of testosterone^72^. Loss of testosterone in males undergoing chemical castration for prostate cancer can result in symptoms comparable to ovariectomy in females, including hot flashes and cognitive decline^73–76^. Further, similar to estrogen, testosterone has been shown to exert a protective effect on mitochondrial bioenergetics and reduce generation of AD pathologies^77,78^, whereas low circulating testosterone level in elder males is associated with increased risk of LOAD^77,79–81^. However, clinical studies on therapeutic effect of testosterone on age-related cognitive decline in males have yielded mixing results^82,83^. An investigative approach comparable to that taken in the current analysis may yield better understanding of the metabolic profile in aging male brain and the impact of testosterone.

We are aware of the limitations of interpreting cross sectional observations to infer longitudinal changes. However, given the type of samples required for this type of mechanistic investigation, there were limited alternatives. While many observed changes did not reach statistical significance, the pattern of changes was consistent and systematic. This is likely due to the use of a natural aging model rather than an accelerated aging or disease model. Using a natural aging model of chronological and endocrinological aging was key to revealing the systems of biology required to respond to energetic demands of brain.

While this study provided systematic and detailed metabolic aging road map in the aging female brain, further hypothesis-driven mechanistic studies are necessary to fully understand the implications of such transitions. The brain is composed of multiple cell types. Neurons primarily rely on glucose as its fuel source^84,85^ whereas astrocytes are the primary source of fatty acid β-oxidation, lactate generation, and the only producer of ketone bodies in brain when glucose availability is limited^86–89^. Given the tight control of supply and demand required for brain bioenergetics, it is important to elucidate how different cell types adapt, communicate with, and support each other. Further, metabolic intermediates from auxiliary fuel sources such as free fatty acids can provoke chronic inflammation, which is also a common theme of age-related neurodegenerative disease. Understanding how the metabolic profile changes correspond with inflammatory markers is key to identifying and deploying effective interventions to promote healthy brain aging.

## Conclusion

The bioenergetic system in the aging brain is complex, adaptive, and dynamic. Chronological aging and endocrinological aging both drive critical aspects of the bioenergetic system in brain. Coupling between brain and peripheral metabolism is dynamic and has implications for therapeutic and nutritional interventions to address brain metabolic distress. Given the parallel metabolic phenotype between aging female brain and prodromal AD, our observations provide insights into preventative and therapeutic windows of opportunity to sustain brain metabolic health and reduce risk of AD.

## Data Availability

The datasets generated during and/or analyzed during the current study are available from the corresponding author on reasonable request.
